# Clinical Markers of Vulnerability for Bleeding in Men With Prostate Cancer and Antithrombotic Treatment

**DOI:** 10.1002/cnr2.70720

**Published:** 2026-10-13

**Authors:** Linda Björkhem‐Bergman, Christel Hedman, Máté Szilcz, Gabriella Frisk

**Affiliations:** ^1^ Department of Neurobiology, Care Sciences and Society (NVS), Division of Clinical Geriatrics Karolinska Institutet Huddinge Sweden; ^2^ Stockholms Sjukhems R&D Department Stockholm Sweden; ^3^ Department of Molecular Medicine and Surgery Karolinska Institutet Stockholm Sweden; ^4^ Department of Clinical Sciences Lund Lund University Lund Sweden; ^5^ Department of Medical Epidemiology and Biostatistics Karolinska Institutet Stockholm Sweden; ^6^ ASIH Stockholm Södra, Advanced Home Care and Specialized Palliative Ward Stockholm Sweden

## Abstract

**Purpose:**

Men with prostate cancer have a high risk for bleeding when treated with antithrombotics. The aim of this study was to investigate clinical markers for vulnerability for bleeding in these men the last year of life.

**Methods:**

This is a retrospective study on deceased patients with advanced prostate cancer admitted to specialized palliative care and treated with antithrombotics the last year of life. Relative risk (RR) was calculated with log‐binomial models.

**Results:**

Of 100 men with end‐stage prostate cancer, 64 men were treated with antithrombotics. Twenty‐four of these (37.5%) suffered from bleeding during the last year of life. Clinical markers associated with an increased vulnerability for bleeding were need of blood transfusion, RR 3.15 (95% CI: 1.34–7.39); low thrombocyte count, RR 2.41 (95% CI: 1.39–4.16); and deep venous thrombosis treatment, RR 2.44 (95% CI: 1.21–4.94). Age, type of antithrombotic, use of steroids, or NSAID were not risk factors. In 50%, antithrombotic treatment continued during the last week in life.

**Conclusions:**

Antithrombotic treatment the last year of life is associated with a high risk of bleeding in men with prostate cancer. Clinical markers associated with increased vulnerability for bleeding are low thrombocyte count and need of transfusions.

## 1 Introduction

Antithrombotic treatments are common in specialized palliative cancer care [1, 2, 3, 4, 5], since patients often benefit from these treatments, regardless of adverse effects [1, 6]. Antithrombotics include antiplatelet drugs and anticoagulants [7]. Clinical guidelines suggest that most patients with cancer and thrombosis benefit from long‐term treatment with antithrombotics [8]. We have recently performed a retrospective study among patients in specialized palliative care, in which we showed a high risk of bleeding in patients using antithrombotics during the last year of life, independent of age, indication for, and type of antithrombotics, while the risk of thrombosis was very low after deprescribing. In this study, patients with advanced prostate cancer had a significantly increased risk of bleeding compared to patients with other cancer types and non‐cancer diagnoses [9]. The primary aim of the present study was to investigate clinical markers of vulnerability for bleeding in the patients with prostate cancer with antithrombotics in the last year of life.

## 2 Methods

### 2.1 Study Design

In this retrospective single‐center cohort study, a review of medical records was performed for all deceased patients who had been admitted to a specialized palliative care unit in Stockholm, Sweden “ASIH Stockholm Södra” [10, 11] from January 1, 2016, until December 31, 2018.

The study was approved by the Swedish Ethical Review Board, Dnr 2018/1798–31.

### 2.2 Study Settings and Participants

The “ASIH Stockholm Södra” unit provides specialized home care and in‐patient specialized palliative care [9]. In the original study, all patients admitted to the unit and deceased between January 1, 2016, until December 31, 2018, were screened for use of antithrombotic (ATC code B01) during the last year of life. Patients with prophylactic antithrombotic treatment after surgery were excluded [9]. In this sub‐study, all patients with prostate cancer with and without antithrombotic were extracted from the original cohort.

### 2.3 Variables

Demographic and clinical data, including date of birth and death, sex, and cause of death were collected from the medical records. Also more specific variables for the cohort as remaining primary tumor (yes/no), bone metastases (yes/no), medication of Non‐Steroidal‐Anti‐Inflammatory‐Drugs (NSAID) the last year of life (yes/no), medication of steroids the last year of life (yes/no), blood transfusion the last year of life (yes/no) and the last blood tests of hemoglobin and thrombocyte counts within 6 months before death were retrieved. Low thrombocyte counts was defined as defined as < 50 × 109/L. For all patients who had been prescribed antithrombotics, the following data were extracted: type of medication, indication for and dose of antithrombotics, date and reason for deprescribing, complications after deprescribing (e.g., thrombosis and stroke), clinical significant bleeding during the last year of life, and date and type of bleeding.

### 2.4 Statistical Methods

Descriptive statistics are presented as *n* (number) and percentage (%). Chi2 test was used for the comparison between the categories. The log‐binomial generalized linear models were used to explore factors associated with bleeding compared to no bleeding. Relative Risks (RR) and 95% confidence intervals (CI) were calculated for each variable potentially related to bleeding: age, indication for antithrombotics, treatment with steroids or NSAID. All these factors were identified a priori based on subject matter knowledge. A reference group was chosen for a comparison for each variable. All analyses were performed with R version 4.3.1.

## 3 Results

### 3.1 Participants

The cohort consists of 100 men with prostate cancer, of whom 64 (64%) were treated with antithrombotics the last year of life. The mean age at death was 79.1 years. The most common antithrombotic treatment was low‐molecular weight heparin (LMWH) in 31 (48.4%) patients, followed by 27 (42.2%) with antiplatelet drugs (aspirin and/or clopidogrel). Only 6 patients (9.4%) had other per oral drugs (warfarin and direct oral anticoagulants (DOACs)). Prophylaxis and atrial fibrillation were the most common indications for antithrombotics. Prophylaxis was defined when no clear indication was found.

### 3.2 Bleeding

Among those with antithrombotics, 24 (37.5%) experienced clinically significant bleeding. The most common sources of bleeding were hematuria for 12 patients, nasal bleeding for four patients, and bleeding from the skin for four patients. One patient had central nervous system (CNS) bleeding.

In the entire cohort, 36 men with prostate cancer had no treatment with antithrombotics. Eight of these men had some form of bleeding (22.2%); the most common was hematuria followed by nasal bleeding. There was no significant difference in risk of bleeding in those treated with antithrombotics compared to those not treated, RR 0.59 (95% CI: 0.29–1.23; *p* = 0.12).

### 3.3 Clinical Markers for Vulnerability for Bleeding

In order to further study the vulnerability for bleeding during antithrombotic treatment, RR was calculated for each variable (Figure 1). Factors associated with increased risk of bleeding were the need for blood transfusion, RR 3.15 (95% CI: 1.34–7.39), and low thrombocyte count, RR 2.41 (95% CI: 1.39–4.16). In addition, those with deep vein thrombosis treatment had a higher risk of bleeding compared to those with prophylactic treatment, RR 2.44 (95% CI: 1.21–4.94). Age, type of antithrombotic, or use of steroids or NSAID were not associated with increased risk.

**FIGURE 1:**
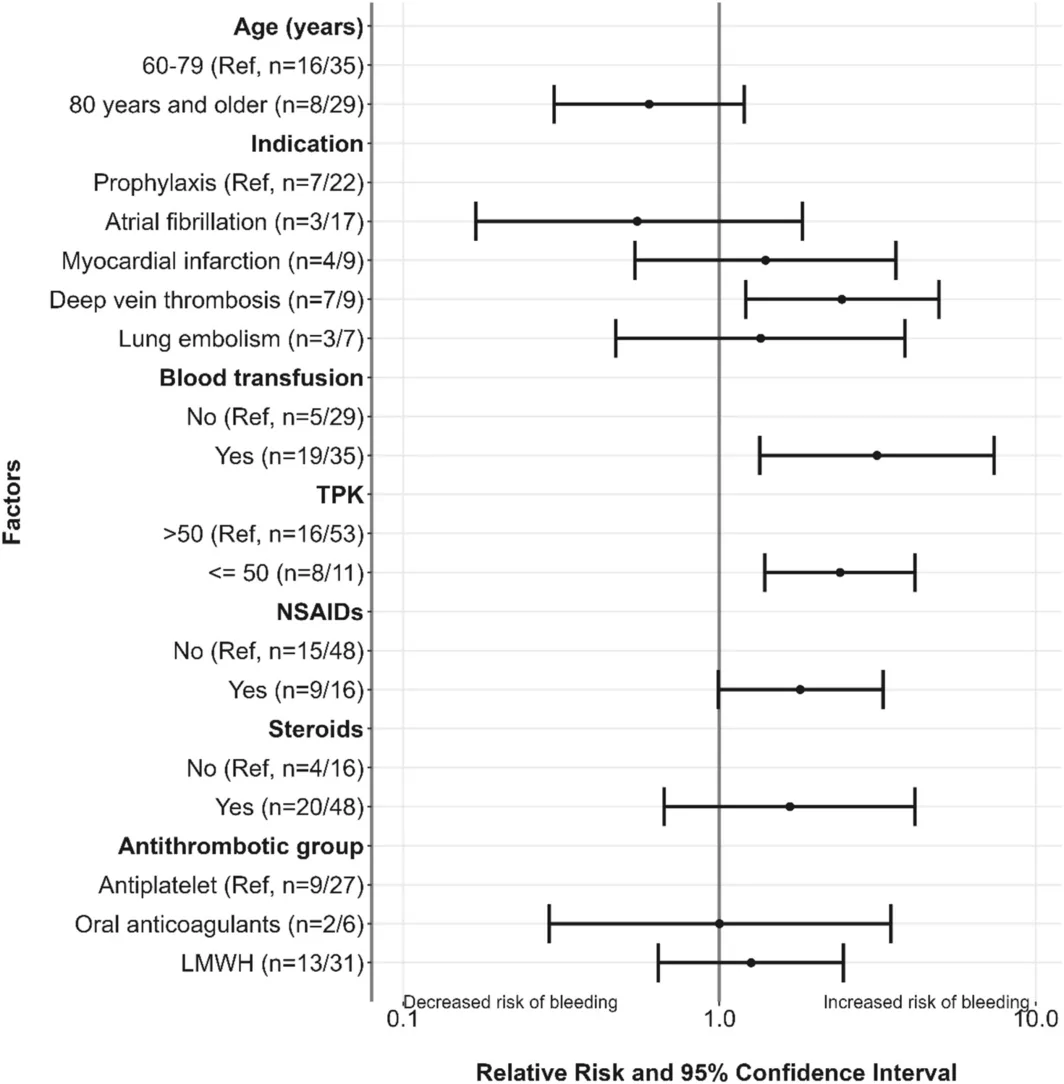
Relative risks (RR) estimates of factors associated with bleeding of men with prostate cancer who used antithrombotic treatments the last year in life. The *x*‐axis displays the variables analyzed for bleeding risk. (*n* = *x*/*N*), where *x* represents the number of individuals with bleeding and *N* denotes the total number of individuals in each group.

### 3.4 Effects of Deprescribing

No patients developed any cardiovascular complications, such as myocardial infarction, stroke, or systemic embolism, after deprescribing antithrombotics during the last year in life.

### 3.5 Timing of Deprescribing

For 32 patients (50%), antithrombotics continued until the last days in life and were deprescribed 0–6 days before death. Antithrombotics were not deprescribed at all for 14 patients (22%). For those who continued treatment until the very last days, approximately 43% received it orally. It is not known whether they were able to swallow the medication, as most patients cannot take oral medications during the last days/day of life.

## 4 Conclusion

To our knowledge, this is the first study that evaluates clinical markers for vulnerability for bleeding in men with prostate cancer and treatment with antithrombotics in palliative care.

Our results show that treatment with antithrombotics in the last year of life for patients with prostate cancer in palliative care is associated with a high risk of bleeding. Clinical markers associated with increased vulnerability to bleeding were need of blood transfusion the last year of life, low thrombocyte count, and if the patient had antithrombotics due to deep vein thrombosis. Moreover, antithrombotics continued during the last week of life for 50% and were not deprescribed at all for 22% of the patients. The difference in risk for bleeding between men with prostate cancer with or without antithrombotics was not statistically significant. However, this might be due to lack of power in this limited cohort. Still, our data do not support a clear increase in bleeding risk attributable to antithrombotics alone.

In the present study, the incidence of bleeding was 37.5% of all men with prostate cancer treated with antithrombotics in the palliative care unit. The incidence of bleeding is higher in the present study than in earlier studies including our original study, where the incidence was 16.1% in all patients treated with antithrombotics [9] and in a French study with Tardy et al. where the incidence of clinically relevant bleeding was 9.8%. However, these studies included patients in palliative care with all kinds of diagnoses, not only men with prostate cancer.

Ajabnoor et al. [12] have previously shown that men with prostate cancer and atrial fibrillation in general have an increased risk for bleeding, which is in line with our earlier study [9] where men with prostate cancer had a higher risk for bleeding compared to other diagnoses.

It is well established that hematuria is common in prostate cancer [13] and it was also the most common source of bleeding in this study. This could be one explanation for the high risk of bleeding in men with prostate cancer, compared to patients with other diagnoses.

Transfusion of blood during the last year of life was a significant risk factor for bleeding. However, this relationship is probably reverse causality, since the bleeding or bone marrow failure might occur before the transfusions. It might, however, be used as a marker for when antithrombotic treatment should be evaluated.

Earlier studies show that high rates of significant bone marrow failure (anemia and thrombocytopenia) in treatment‐refractory patients with prostate cancer predict an unfavorable prognosis [14]. This is in concordance with our results showing that low thrombocyte count was associated with a high risk of bleeding. Thus, when thrombocytopenia occurs, it might be time to evaluate treatment with antithrombotics.

This study has several limitations to be mentioned. First, the sample size was small, which may affect the results in terms of power, and the effect estimates should be interpreted with caution. The confidence intervals are wide, and there is high potential for both type I and type II errors. Second, the study is based on retrospective data and confounding factors, and reverse causality might be present. Thus, no causal relationships can be drawn. Due to the small sample size and retrospective, non‐interventional design, no firm conclusions about safety after describing can be drawn. This needs to be evaluated in a future prospective study. Moreover, laboratory values were obtained up to 6 months before death and may not reflect the status at the time of bleeding and might be a source of misclassification. Furthermore, hematuria is common in patients with prostate cancer, regardless of whether they are treated with antithrombotics or not. Finally, the results are based on data from a single Swedish specialized palliative care unit. Thus, the results may not be generalized to other healthcare systems or less advanced care settings.

The results from this study might be used for the design of a future, prospective study for evaluating the effects and safety of describing antithrombotics.

To conclude, antithrombotic treatment the last year of life is associated with a high risk of bleeding in men with prostate cancer. Clinical markers associated with increased vulnerability for bleeding are low thrombocyte count and need for transfusions. Given the increased risk of bleeding and the often limited life expectancy, the potential benefits of preventing thromboembolic events must be weighed against the potential harms of antithrombotic treatment in each case.

## Author Contributions

**Linda Björkhem‐Bergman:** conceptualization, funding acquisition, methodology, validation, writing – review and editing, supervision, resources. **Christel Hedman:** conceptualization, methodology, validation, writing – review and editing. **Máté Szilcz:** software, formal analysis, visualization, validation, writing – review and editing, methodology. **Gabriella Frisk:** conceptualization, investigation, writing – original draft, methodology, validation, writing – review and editing, project administration, data curation.

## Funding

This study was financially supported by grants from the Swedish Cancer Society (CAN2018/316), The Swedish Research Council (2022‐00651), Stockholm County Council (FoUI‐974833) and Center for Innovative Medicine (CIMED).

## Conflicts of Interest

The authors declare no conflicts of interest.

## Data Availability

The data that support the findings of this study are available from the corresponding author upon reasonable request.
